# Effects of Sleep Disorders and Circadian Rhythm Changes on Male Reproductive Health: A Systematic Review and Meta-analysis

**DOI:** 10.3389/fphys.2022.913369

**Published:** 2022-07-13

**Authors:** Ou Zhong, Biyun Liao, Jinyuan Wang, Ke Liu, Xiaocan Lei, Linlin Hu

**Affiliations:** ^1^ Reproductive Medicine Center, The Affiliated Hospital of Youjiang Medical University for Nationalities, Baise, China; ^2^ Clinical Anatomy and Reproductive Medicine Application Institute, Hengyang Medical School, University of South China, Hengyang, China

**Keywords:** sleep disorders, sleep quality, circadian rhythms, male reproduction, semen parameters

## Abstract

**Objectives:** The purpose of this study was to elucidate the relationship between sleep disorders and male reproductive health, and to explore the underlying mechanisms via a systematic review and meta-analysis.

**Methods:** PubMed, Embase, The Cochrane library, Web of Science, Scopus databases were searched to collect clinical research on the effects of sleep disorders on male semen parameters from inception to February 24, 2022. RevMan 5.4 was used for meta-statistical analysis. Stata16 software was used to detect publication bias.

**Results:** The results of meta-analysis showed that sleep disorders were associated with reduced total sperm count (mean difference (MD) = −27.91, 95% CI = (−37.82, −18.01), *p* < 0.001), reduced sperm concentration (MD = −5.16, 95% CI = (−9.67, −0.65), *p* = 0.02), reduced progressive motility (MD = −2.94, 95% CI = (−5.28, −0.59), *p* = 0.01), and reduced normal morphology (MD = −0.52, 95% CI = (−0.80, −0.24), *p* < 0.001). However, there is no significant association between sleep disorders and semen volume/reproductive hormones. Further bioinformatics mining revealed that related clock genes (PER1, PER2, CRY2, NR1D1 and NPAS2) were down-regulated in non-obstructive azoospermia patients.

**Conclusion:** In conclusion, current evidence suggests that sleep disorders have a negative impact on male reproductive health, and its underlying mechanism may be related to circadian rhythm disorders. However, the relationship between sleep disorders and reproductive hormone levels has not been found. Due to the limited number and quality of included studies, the above findings need to be validated by more high-quality studies.

## Introduction

Infertility is considered as a public health epidemic. The World Health Organization (WHO) estimates that 9% of couples worldwide struggle with infertility, and that impaired male reproduction contributes to 50% of the issues (Du et al., 2020a). Since the 1990s, studies in several countries and regions around the world have suggested that the quality of male semen is declining year by year and that the decrease in semen quality is an important factor in the increasing incidence of infertility (Carlsen et al., 1992; Cooper et al., 2010; Huang et al., 2017). Therefore, exploring the factors associated with human semen damage can help in the development of preventive and therapeutic measures, which are important for maintaining the reproductive health of the population. In recent years, in addition to concerns about the impact of environmental, dietary and obesity factors on semen quality, the impact of changes in lifestyle and behaviors on sperm quality is gradually attracting attention (Liu et al., 2020).

Sleep is a natural, cyclical state of rest for the brain and body, occurring at regular intervals and regulated by circadian rhythms. Sleep disorder is an abnormal sleep duration or abnormal behavior in sleep caused by a variety of reasons including insomnia and parasomnias (Latreille, 2019). From 1985 to 2012, the report shows that the percentage of US adults who sleep <7 h on average has almost doubled to about 33% (Ford et al., 2015). In 2015, the American Academy of Sleep Medicine and the Sleep Research Society issued a consensus statement stating that 7.0–9.0 h of sleep is most beneficial to the health of adults (18–60 years), whereas fewer than 6.0 h of sleep was insufficient, however, there is no consensus on the value or harm of sleeping more than 9.0 h (Watson et al., 2015). In addition, shift work can undermine sleep quality by interfering with the body’s natural circadian rhythm. One study showed that shift work was an independent risk factor for poor sleep quality (McDowall et al., 2017). Inadequate sleep and shift work is associated with adverse health outcomes including all-cause mortality, cardiovascular disease, hypertension and diabetes (Bara and Arber, 2009; Gamble et al., 2013; Shan et al., 2015; Kim et al., 2018; Domínguez et al., 2019; Wang et al., 2019; Chen et al., 2020). Similarly, inadequate sleep duration and poor sleep quality are potential risk factors for reduced semen quality. Several studies have investigated the relationship between sleep disorders and semen quality in men, however, the results have been inconsistent (Jensen et al., 2013; Li et al., 2013; Liu et al., 2017; Du et al., 2020b; Huang et al., 2021). This study collects current research to investigate the relationship between nocturnal sleep disorders and semen quality parameters in men and analyses gene expression in non-obstructive azoospermia (NOA) patients to explore the impact of sleep disorders and biological clock disorder on male reproductive health.

## Materials and Methods

### Search Strategy

Clinical research on the effects of sleep disorders on male semen parameters were found by searching PubMed, Embase, The Cochrane Library, Web of Science, and Scopus databases. The search was conducted using a combination of subject and free words. Search terms include: “Sleep”, “sleep*", “sleep disturbance”, “Sleep Disorders”, “insomnia”, “dyssomnia”, “Sleep Deprivation”, “circadian rhythm”, “sleep duration”, “bedtime”, “sleep quality”, “shift work”, “night work”, “Reproducible health”, “reproduction”, “sperm count”, “seven parameters”, “sperm*", “seven*", “seven quality”, “reproducible hormones”, “fold stimulating hormone”, “luteinizing hormone”, “testosterone”, etc. For a complete list of search terms, see Supplementary Appendix SA1. The most recent search was performed on February 24, 2022.

### Inclusion and Exclusion Criteria

The inclusion criteria were as follows: 1) experimental group (poor sleep): including sleep <7 h, bedtime >24:00, night shift work; control group (good sleep): including sleep >7 h, bedtime <24:00, non-shift work; 2) study type: cohort study, case-control study, cross-sectional study. The exclusion criteria were as follows: 1) studies with patients with BMI >30 kg/m^2^, age >50 years old, or sample size <10; 2) patients included in the study were not excluded from relevant reproductive disorders including cryptorchidism, orchitis, epididymitis, sexually transmitted diseases, etc; 3) missing semen parameters data; 4) case-series/reports, expert opinions, randomized controlled trials, conference abstracts and review articles; 5) animal experiments, cell experiments and other articles without available data; and 6) articles of poor quality and with significant statistical errors. The details for inclusion were shown in Supplementary Appendix SA2.

### Literature Screening and Data Extraction

Following the retrieval of the literature, at least two individuals independently reviewed the title, abstract, and full text of the articles base on the inclusion and exclusion criteria. Firstly, duplicate articles were weeded out, and then titles and abstracts were read. Following the exclusion of articles with plainly unrelated topics, the whole text was examined again to establish whether the articles were included or not, and data extraction for the included articles was performed.

### Quality Assessment

The methodological quality of the included cohort study was assessed with the Ottawa–Newcastle scale using a star-based system, which analyzes the study groups’ selection, comparability, and ascertainment of the outcome of interest (Stang, 2010). Studies with a score of >7 had a low risk of bias, those with a score of 5–7 had a moderate risk of bias, and those with a score of <5 had a high risk of bias.

### Indicators

The total sperm count (million), the sperm concentration (million/ml), semen volume (ml), progressive motility (%) and Normal morphology (%) were defined as primary outcomes in this study. The serum levels of Reproductive hormones (testosterone, LH and FSH) were defined as the secondary outcomes in this study.

### Gene Expression Profile and Differential Gene Expression Analysis

Gene expression data from the GSE45887 dataset, based on the GPL6244 platform (Affymetrix Human Gene 1.0 ST Array), was downloaded from the Gene Expression Omnibus (GEO) database (Barrett et al., 2013). GSE45887 included genes expression profiles from testes from four patients with non-obstructive azoospermia (NOA) and four patients with normal spermatogenesis. GEO2R was applied to identify differentially expressed genes (DEGs) between the NOA and normal samples, based on |log fold change (FC)| > 0 and P.Value <0.05. We also intersected the DEGs with clock genes and applied the pheatmap package to plot the Heatmap of differentially expressed clock genes.

### Statistical Analysis

RevMan 5.4 was used for meta-statistical analysis. Continuous data were calculated with weighted mean difference (MD), and confidence intervals (CIs) were set at 95%; *p* < 0.05 was considered statistically significant. Testosterone was calculated using Standardized Mean Difference (SMD) because of the different data units. Heterogeneity between studies was evaluated by I^2^ and *p* values. If I^2^ > 50% and *p* < 0.1, heterogeneity across studies was considered to be existed, and the SMD and MD was calculated using the random-effects model; otherwise, the fixed-effects model was applied. SMD and MD for all primary and secondary outcomes were calculated, using the random effect model due to the significant heterogeneity in the included studies. Stata16 software was used to detect publication bias, Egger and Begg methods were mainly used, *p* > 0.05 indicates no significant publication bias (because Egger examination is more sensitive, when the two results are contradictory, the Egger examination results are given priority).

## Results

### Study Selection

**FIGURE 1:**
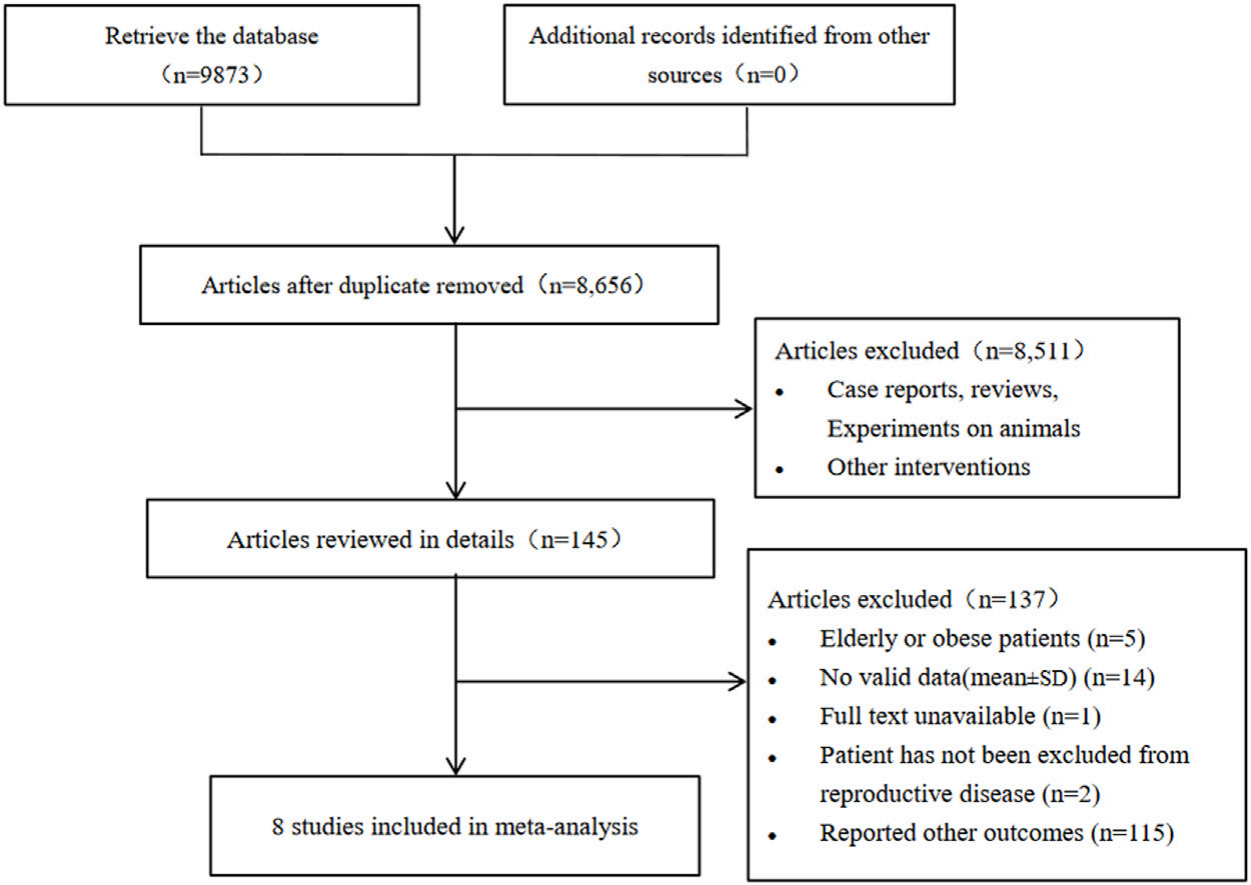
Flowchart of study selection.

A total of 9873 articles were identified in the initial retrieval including PubMed (*n* = 296), Embase (*n* = 1218), The Cochrane library (*n* = 1067), Web of Science (*n* = 4015) and Scopus (*n* = 3277). Of these, 1215 duplicate articles were excluded after carefully examining the titles and abstracts. After further screening, eight studies were included in the meta-analysis, the literature screening process and results were shown in Figure 1. Eight studies were eventually included in this study, including one cohort study and seven cross-sectional studies. A total of 2169 patients with sleep disorders and 3858 control patients with normal sleep were enrolled, all aged less than 50 years and with a BMI of less than 30 kg/m^2^. The clinical characteristics of the included studies were shown in Table 1.

**TABLE 1:** Characteristics of included studies.

Study	Country	Population Size (E/C)	Characteristic (E/C)	Experimental Group (Poor Sleep)	Control Group (Good Sleep)	Newcastle ottawa
Bara and Arber, (2009)	China	218/1439	Age 22.5 ± 3.7 (all)	Late bedtime (>24:00)	Normal bedtime (22:00–23:00)	Cross-Sectional study
			BMI 21.7 ± 2.8 (all)			
Barrett et al. (2013)	Turkey	104/116	Age 30.9 ± 5.0/32.9 ± 5.07	Shift working group	Non-shift working group	Cross-Sectional study
			BMI 26.59 ± 4.57/28.44 ± 19.75			
Liu et al. (2020)	China	856/321	Age 32.60 ± 5.71/30.30 ± 4.96	sleep duration 4–6 h	sleep duration >8 h	Cross-Sectional study
			BMI 23.86 ± 3.71/23.23 ± 3.45			
Huang et al. (2021)	China	378/592	Age 31.63 ± 5.87/31.90 ± 5.97	sleep duration 6.86 ± 1.02	sleep duration 7.69 ± 0.79	Cross-Sectional study
			BMI 24.60 ± 4.02/23.76 ± 3.36			
Huang et al. (2019)	United States	75/96	Age 36.3 ± 5.4/36.1 ± 5.7	Shift working group	Non-shift working group	Cross-Sectional study
			BMI -			
Mishra et al. (2018)	China	45/81	Age 20 (20–21)	sleep duration ≤6.5 h	sleep duration 8.5–9 h	Cross-Sectional study
			BMI 20.9 (19.6–22.7)			
Mishra et al. (2018)	China	45/148	Age 20 (20–21)	sleep duration ≤6.5 h	sleep duration 8–8.5 h	Cross-Sectional study
			BMI 20.9 (19.6–22.7)			
Mishra et al. (2018)	China	45/187	Age 20 (20–21)	sleep duration ≤6.5 h	sleep duration 7.5–8 h	Cross-Sectional study
			BMI 20.9 (19.6–22.7)			
Liu et al. (2017)	China	13/328	Age 20–40	sleep duration <4 h	sleep duration >8 h	Cross-Sectional study
			BMI -			
Liu et al. (2017)	China	180/328	Age 20–40	sleep duration 4–6 h	sleep duration >8 h	Cross-Sectional study
			BMI -			
Du et al. (2020b)	China	106/111	Age 30.25 ± 5.18/30.18 ± 5.13	sleep duration <6 h	sleep duration 7–8 h	prospective cohort study
			BMI -			(Newcastle Ottawa:8; Follow-up:6 months)
Du et al. (2020b)	China	104/111	Age 29.84 ± 4.89/30.18 ± 5.13	Late bedtime (>24:00)	Normal bedtime (22:00–24:00)	prospective cohort study
			BMI -			(Newcastle Ottawa:8; Follow-up:6 months)

Effects of sleep disorders on total sperm count, sperm concentration, semen volume, progressive motility, and normal morphology and reproductive hormone.

**FIGURE 2:**
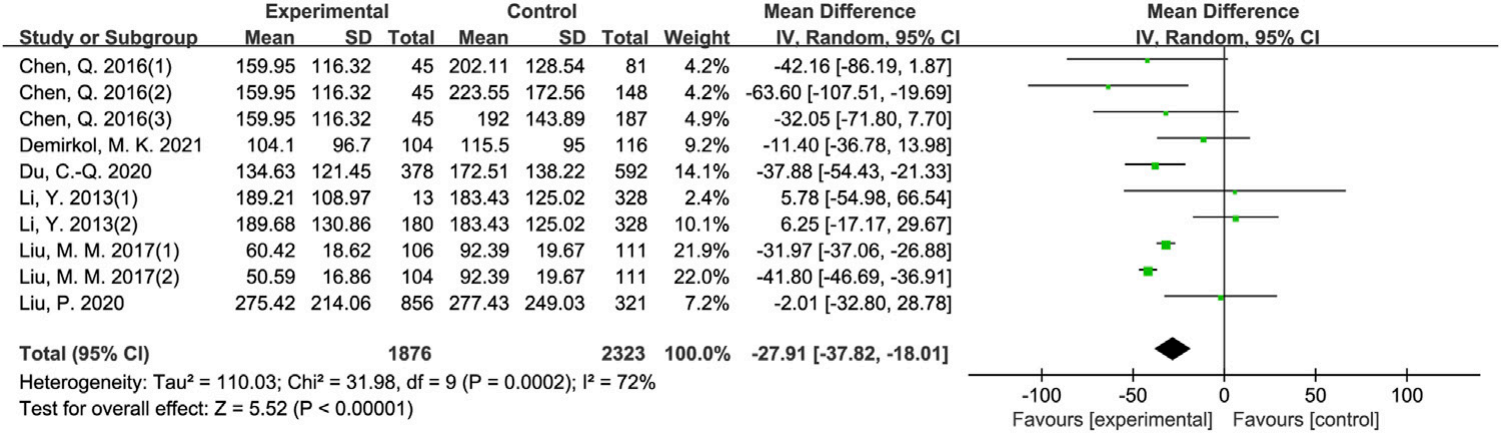
Forest map evaluating the effects of sleep disorders on total sperm count.

**FIGURE 3:**
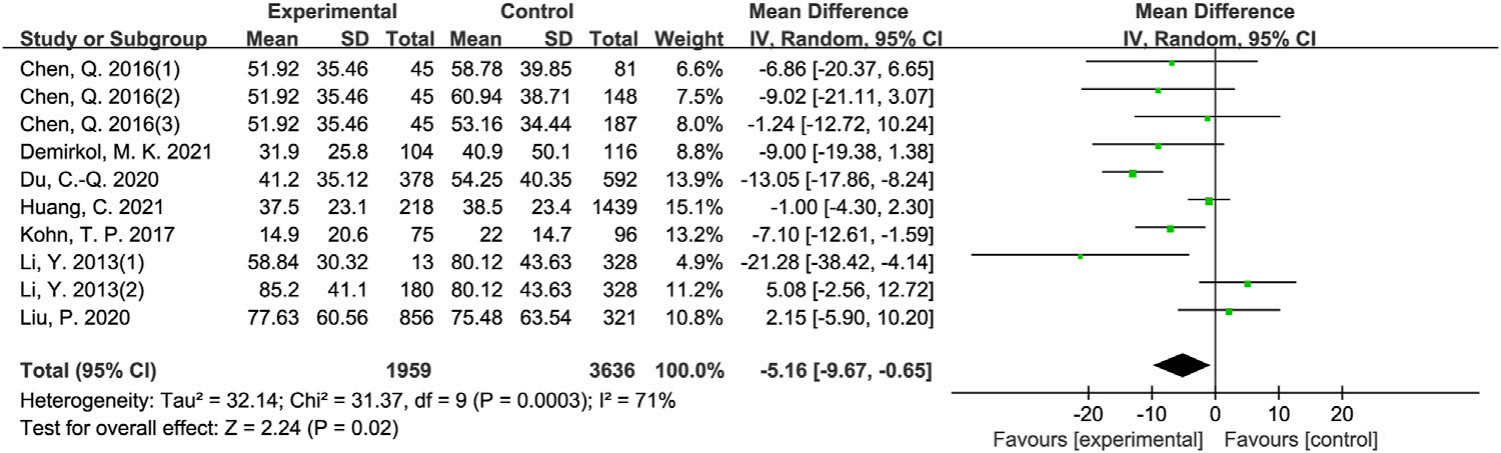
Forest map evaluating the effects of sleep disorders on sperm concentration.

**FIGURE 4:**
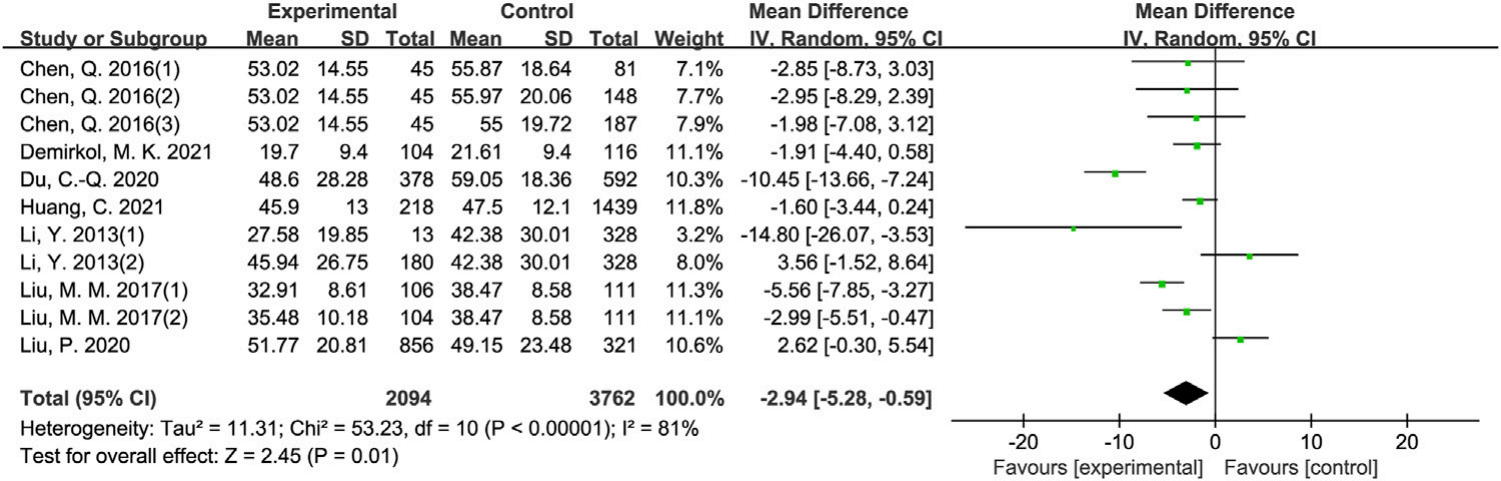
Forest map evaluating the effects of sleep disorders on progressive motility.

**FIGURE 5:**
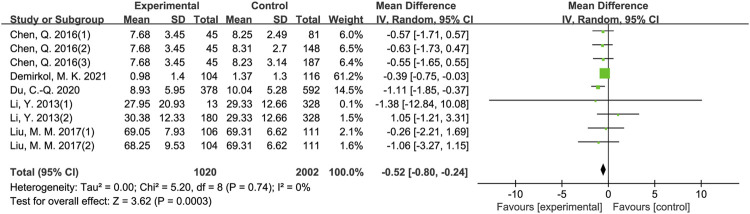
Forest map evaluating the effects of sleep disorders on normal morphology.

**FIGURE 6:**
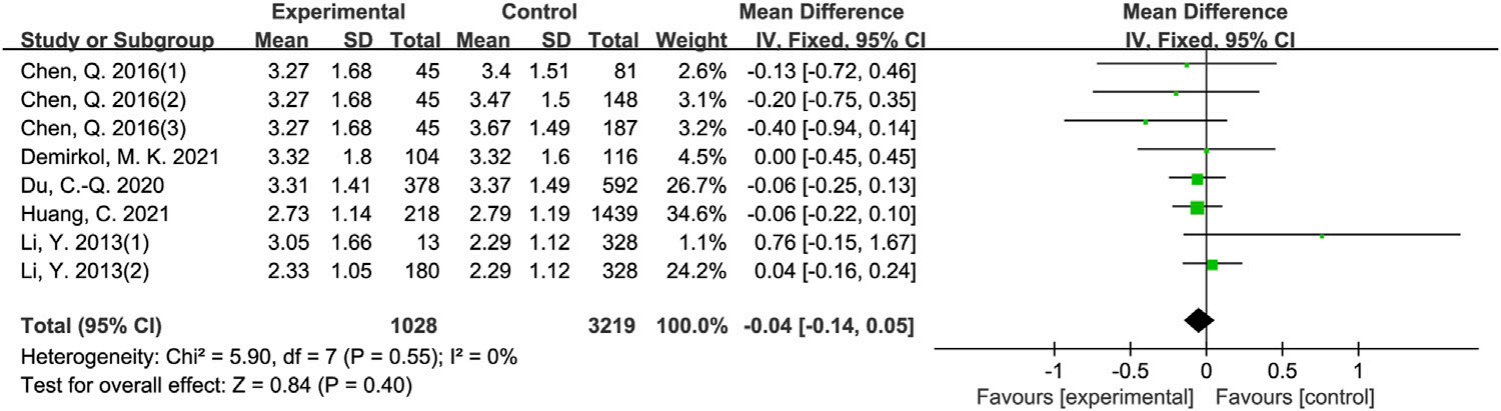
Forest map evaluating the effects of sleep disorders on semen volume.

**FIGURE 7:**
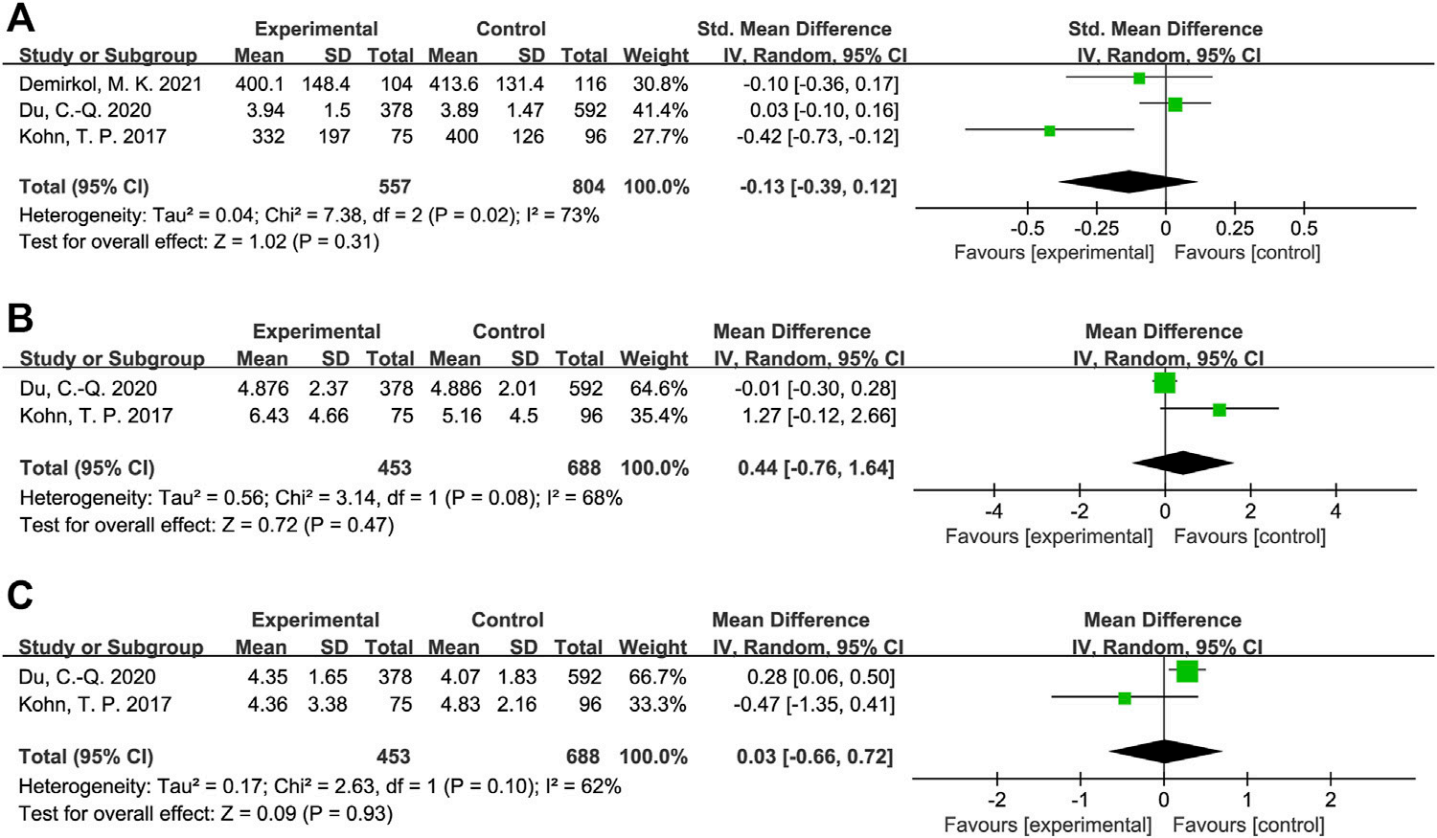
Forest map evaluating the effects of sleep disorders on testosterone **(A)**, FSH **(B)** and LH **(C)**.

The results of meta-analysis showed that patients from sleep disorders group were associated with reduced total sperm count (MD = −27.91, 95% CI = (−37.82, −18.01), *p* < 0.001; Figure 2), reduced sperm concentration (MD = −5.16, 95% CI = (−9.67, −0.65), *p* = 0.02; Figure 3), reduced progressive motility (MD = −2.94, 95% CI = (−5.28, −0.59), *p* = 0.01; Figure 4), and reduced normal morphology (MD = −0.52, 95% CI = (−0.80, −0.24), *p* < 0.001; Figure 5) when compared to the control group. However, sleep disorders was not significantly associated with semen volume (MD = −0.04, 95% CI = (−0.14, −0.05), *p* = 0.40; Figure 6), testosterone (SMD = −0.13, 95% CI = (−0.39, −0.12), *p* = 0.31; Figure 7A), FSH (MD = 0.44, 95% CI = (−0.76, 1.64), *p* = 0.47; Figure 7B) and LH (MD = 0.03, 95% CI = (−0.66, 0.72), *p* = 0.93; Figure 7C) when compared to the control group. No significant publication bias was found in the results of Begg’s plots and Egger’s test for total sperm count (*p* = 1.8926/0.6642), sperm concentration (*p* = 1.6289/0.1639), progressive motility (*p* = 1.7871/0.2992) and normal morphology (*p* = 0.9170/0.8881).

### Differentially Expressed Clock Genes

**FIGURE 8:**
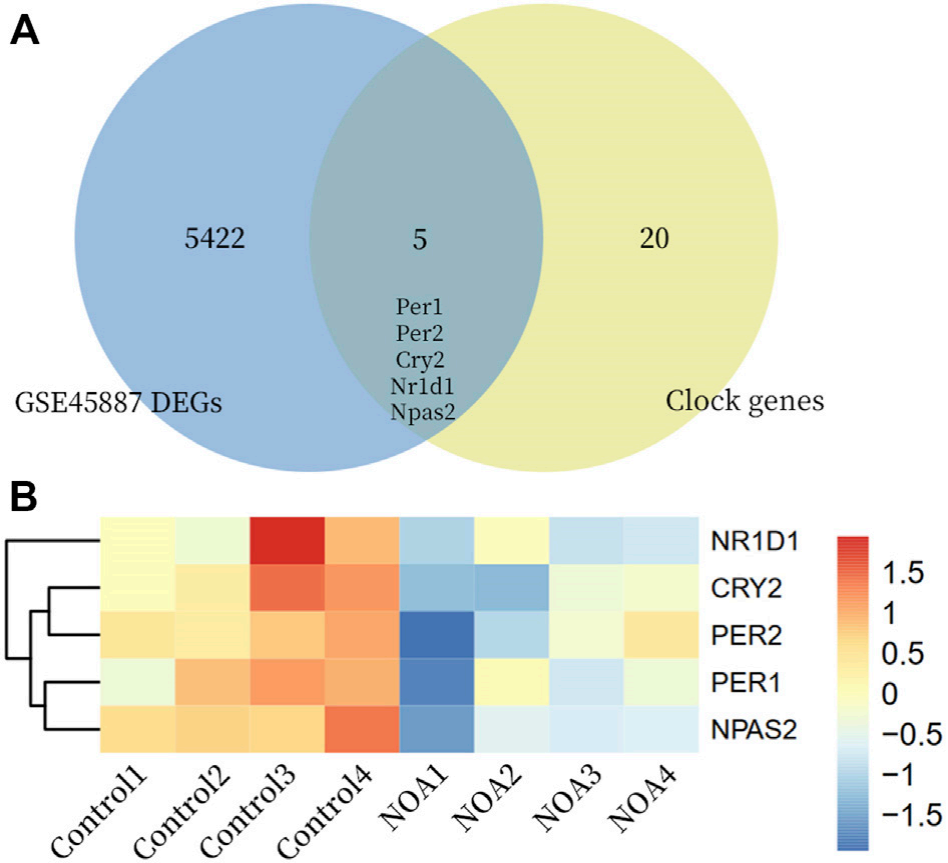
Differential clock genes. **(A)** Venn diagrams of GSE45887 gene set intersecting clock genes; **(B)** Heat map of differential clock genes.

Gene expression profiles in testicular tissues of 4 NOA patients and 4 normal spermatogenesis patients were retrieved from the GSE45887 dataset in NCBI-GEO database. Based on |logFC >0| and *p* < 0.05, 5542 DEGs were identified. We cross-identified with currently known biological clock genes and identified five differentially expressed clock genes (Table 2; Figure 8).

**TABLE 2:** Clock genes obtained after crossover.

Gene Symbol	*p* value	logFC
PER1	2.00E-02	−0.999762
PER2	3.00E-02	−0.5701285
CRY2	1.21E-02	−0.568914
NR1D1	2.68E-02	−0.9292505
NPAS2	4.91E-04	−1.0373125

### Sleep Disorders Affect the Quality of Sperm

**FIGURE 9:**
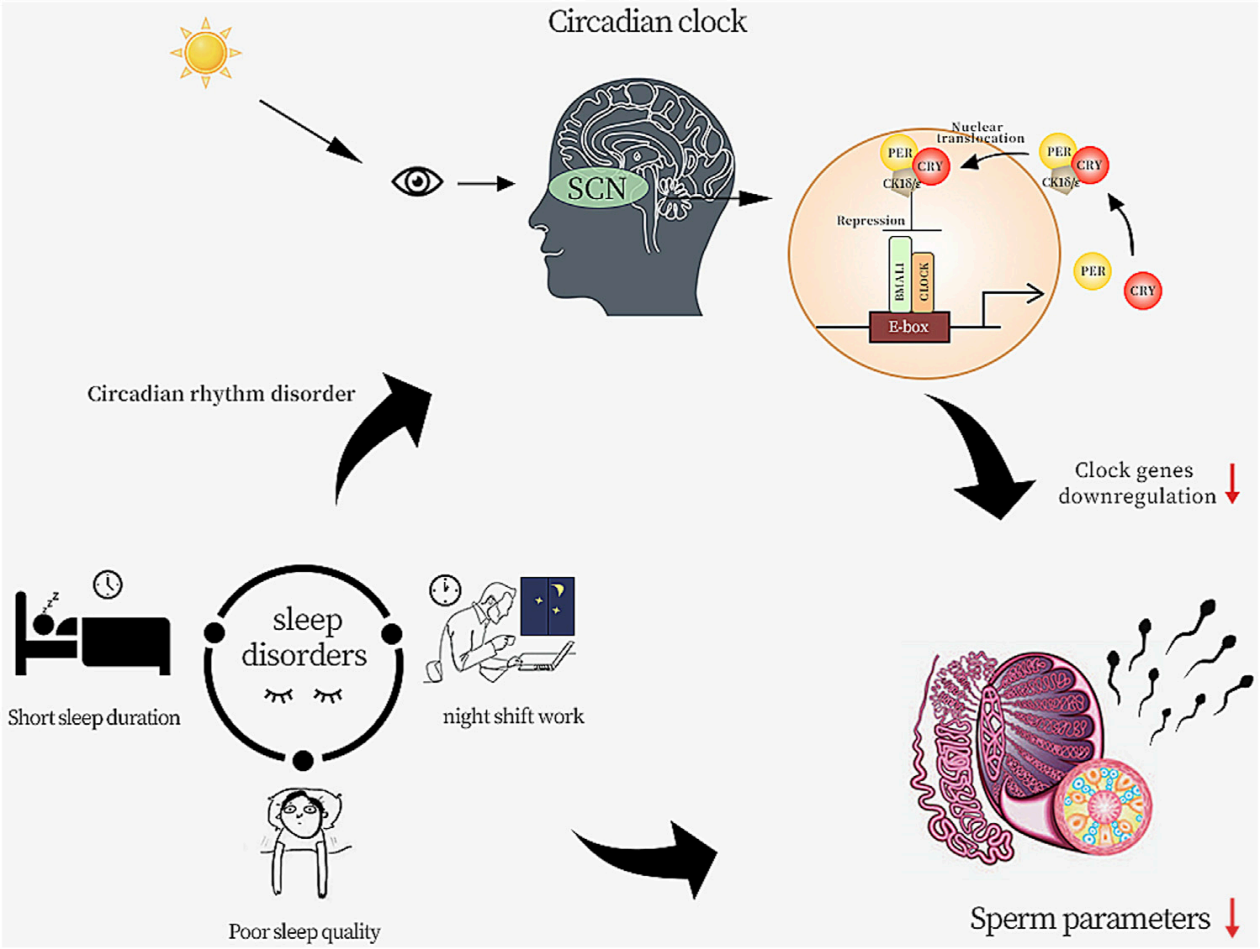
Sleep disorders affect the quality of sperm.

As shown in Figure 9, in the core feedback loop of the mammalian biological clock, CLOCK (orange circle) and BMAL1 (green circle) binds the E-box enhancer elements of the Per and Cry genes and stimulates their expression. PER (yellow circle) and CRY (red circle) dimerize and translocate to the nucleus after binding with casein kinase 1δ (CK1δ) or CK1ε, where they repress their own transcription.

## Discussion

In recent years, studies in several countries have shown that the quality of male semen is decreasing year by year (Jensen et al., 2013; Mishra et al., 2018; Huang et al., 2019). With the rapid development of the economy and the acceleration of the pace of life, the incidence of sleep disorders has become more and more prominent in the population, and has seriously endangered people’s physical and mental health, and its impact on semen quality has gradually attracted the attention of scholars. In this study, we included eight studies, which included 2,169 patients with sleep disorders and 3,858 patients with normal sleep. The results of the meta-analysis showed that sleep disorders were associated with reduced total sperm count, reduced sperm concentration, reduced progressive motility, and reduced normal morphology. However, there is no significant association between sleep disorders and semen volume and reproductive hormones. In a study of 796 University students, there was an inverted U-shaped relationship between sleep duration and sperm volume and total sperm count (Chen et al., 2016). Similarly, Wise et al. reported that short and long sleep were associated with reduced fertility in couples as an important consequence of low semen quality (Shi et al., 2020). The mechanisms underlying the relationship between sleep disorder and semen quality are unclear. Studies have shown that sleep disorder can lead to decreased testosterone levels (Leproult and Van Cauter, 2011a), disorder of circadian rhythm (Gamble et al., 2013), elevated inflammatory or pro-inflammatory cytokines (Haack et al., 2007; Hærvig et al., 2018), decreased antioxidant capacity of the body (Torres et al., 2014), and disruptions in the activity of enzymes associated with sperm apoptosis (e.g. nitric oxide synthase and endothelial-type nitric oxide synthase) (Middendorff et al., 1997; O'Bryan et al., 2000), ultimately leading to impaired male reproductive health. Animal experiments have found that sexually mature male rats subjected to sleep deprivation exhibit not only reduced sexual function and sperm motility, but also decreased testosterone levels (Wu et al., 2011). In addition to animal experiments, sleep restriction was shown in a study to alter sex hormone levels in 10 healthy male volunteers who underwent continuous sleep restriction for a week (5 h of sleep per night) and whose testosterone levels were significantly reduced (Leproult and Van Cauter, 2011b). Sleep deprivation may inhibit testosterone synthesis by altering the expression of iNOS to induce oxidative stress, upregulating 5-hydroxytryptamine expression and inhibit steriodogenic acute regulatoryprotein (stAR) activity (Wu et al., 2011; Chouhan et al., 2015). However, Du et al. documented a negative association between overall sleep quality and several semen parameters, but similar to previous studies similarly failed to find a significant association between sleep duration and reproductive hormone levels (FSH, LH, estradiol, progesterone, testosterone and prolactin) (Chen et al., 2016; Du et al., 2020b). In this study, again, no association between sleep disorders and reproductive hormones could be found due to the limitations of the sample size. In addition to this, we conducted a subgroup analysis of different types of sleep disorders and showed that shift work was associated with reduced sperm concentration, late sleep was associated with reduced total sperm count and progressive motility, and short sleep duration was associated with reduced normal morphology. Studies have shown that age is also an important factor in the decline of semen quality (Verón et al., 2018). In this study, we conducted subgroup analysis for different age stages, but due to the limited sample size, we did not find a role for age in this study.

Inappropriate sleep habits can disrupt the expression of circadian genes, which can have a detrimental effect on the male reproductive system (Boden et al., 2013; Gamble et al., 2013). The mammalian circadian system consists of a central circadian pacemaker in the hypothalamic suprachiasmatic nucleus (SCN) and peripheral clocks in other brain regions and tissues throughout the body, including liver, muscle, adipose tissue, and testes. The SCN receives information about the 24-h light-dark cycle through the retina, and this timing signal is relayed to the peripheral biological clock via neural and hormonal signals and body temperature to maintain homeostasis in the organism (Alvarez et al., 2003; García-Costela et al., 2020). The circadian clock is an endogenous mechanism that governs 24-h rhythmic variation in organism behaviour and physiology, which is regulated by a core set of clock genes that coordinate their transcriptional and translational autoregulatory feedback loops at the cellular level (Figure 9). The core clock genes include Clock, Arntl, period (Per)1, Per2, Per3, cryptochrome (Cry)1, and Cry2, which are necessary for generating and maintaining circadian rhythmicity (Boden et al., 2013; Li et al., 2018; Zhang et al., 2022). In recent years, there has been an increasing recognition of the importance of clock genes and circadian rhythm regulation for health; biological clock genes have been directly linked not only to sleep disorders but also to diabetes, cancer, bipolar disorder, and infertility (Takahashi et al., 2008; Welsh et al., 2010; Hodžić et al., 2013; Sciarra et al., 2020). We analyzed testicular specimens from four patients with non-obstructive azoospermia (NOA) versus four patients with normal spermatogenesis and found that the clock genes (Per1, Per2, Cry2, Nr1d1 and Npas2) were significantly downregulated in NOA patients. A study by Zhang, P. et al. demonstrated similar content, finding that the expression levels of five clock genes (BMAL1, Clock, CRY1, PER1 and PER2) were significantly lower in the sperm of infertile men with weak spermatozoa (AZS) than in normal fertile men (Zhang et al., 2022). Liang et al. downregulated clock gene expression in male mouse testes and found lower *in vitro* fertilization rates in clock knockout sperm, lower blastocyst formation rates, lower acrosome enzyme activity and delayed oocyte dispersal. These results suggest that acrosome enzyme activity can be regulated by the clock and that the clock contributes to the regulation of male fertility and blastocyst formation (Liang et al., 2013). The clock proteins have been reported to be expressed in the male germ epithelium. CRY1 is distributed in the Sertoli cells, spermatogonia, spermatocytes, and the interstitial area. Studies have shown that Cry1-deficient mice have increased numbers of degenerating and apoptotic germ cells in the testis, and corresponding to lower epididymal sperm counts (Li et al., 2018). Knockdown of Bmal1 in sperm leads to sterility in mice, which is manifested by the induction of reduced expression of the StAR gene and related proteins, resulting in low testosterone levels and high serum LH levels, suggesting Leydig cell dysfunction (Alvarez et al., 2008). These results demonstrate the potential role of biological clock in regulating reproductive metabolism.

This study is the first known meta-analysis of sleep and male reproductive health and demonstrates that sleep disorders can affect sperm parameters. However, there are limitations in this study: 1) Most of the studies included in this study were cross-sectional, we were unable to determine the causal direction of the effect, and the results of the cross-sectional studies will be less reliable than those of the cohort studies, and the effect of some other factors cannot be excluded. 2) Some participants were recruited from the fertility institute rather than the general population, which increases the potential for selection bias. 3) Although most studies will exclude subjects with certain male reproductive system diseases, a thorough screening of male diseases has not been carried out, thus, the number of diseases screened by different studies may cause some bias. 4) In addition, some of the patients included had a history of smoking or alcohol consumption, the effect of which on sperm quality could not be excluded. 5) The large variation in sample size of the included studies may have led to some heterogeneity.

## Conclusion

In conclusion, current evidence suggests that sleep disorders have a negative impact on male reproductive health, and its underlying mechanism may be related to circadian rhythm disorders. However, the relationship between sleep disorders and reproductive hormone levels has not been found. Due to the limited number and quality of included studies, the above findings need to be validated by more high-quality studies.

## Data Availability

The original contributions presented in the study are included in the article/Supplementary Material, further inquiries can be directed to the corresponding authors.
