# Supplementary material for: Effects of Sleep Disorders and Circadian Rhythm Changes on Male Reproductive Health: A Systematic Review and Meta-analysis
Source: Front Physiol. 2022 Jul 13;13:913369. doi: 10.3389/fphys.2022.913369 (PMC9326175; doi:10.3389/fphys.2022.913369)
Supplement: Supplementary file 1 [file DataSheet2.docx]

| Huang, C. 2021 | The guidelines were as follows : (i) donors must be between 22 and 45 years of age; (ii) donors must be in good health, based on the results of a physical examination and a psychological evaluation by a qualified doctor, and have no familial history of a genetic disease; (iii) fresh semen should have a liquefaction time of < 60 min, sperm concentration ≥60×10^6^/ml, progressive sperm motility ≥60%, and percentage of normal morphology >30%; (iv) post-thaw semen should have a motility of ≥40%, ≥12×10^6^ motile spermatozoa per vial, and a frozen-thaw survival rate ≥60%; and (v) potential donors must undergo laboratory testing to exclude individuals at high risk for sexually transmitted infections and genetic diseases, including HIV-1 and HIV-2, hepatitis B and C, syphilis, gonorrhea, mycoplasma, chlamydia, cytomegalovirus, Toxoplasma gondii, rubella virus, herpes simplex virus types 1 and 2, and undergo a karyotype analysis. |
| --- | --- |
| Demirkol, M. K. 2021 | Those with azoospermia, severe oligospermia (sperm concentration <1×10⁶/ml), systemic diseases such as DM, cancer, urinary tract infection and febrile illnesses, and history of epididymo-orchitis, sexually transmitted diseases and history of testicular torsion, undescended testes and varicocele surgery were excluded (total 85 men). Subsequently, 220 men agreed to participate in the study by giving informed consent in accordance with the Declaration of Helsinki. |
| Liu, P. 2020 | Inclusion criteria: (i) male aged 18-60 years; (ii) abstinence time of 2-7 d required, complete semen sample collection, no missing.  Exclusion criteria: (i) missing sperm quality data; (ii) patients with cryptorchidism, orchitis, epididymitis, venereal disease, azoospermia; (iii) patients with past history of chronic diseases and long-term use (more than 3 months of continuous use) or recent use (within the last 1 month) of drugs affecting semen quality (including but not limited to spermogenic capsules, and related conditioning traditional Chinese Medicine). |
| Du, C.-Q. 2020 | A total of 1536 volunteers were examined from October 2017 to July 2019, and included in the present study. Among them, 566 were excluded from the analysis due to the following reasons: 135 had reproductive disorders (e.g. varicocele, hydrocele, cryptorchidism, orchitis, testicular torsion) or other chronic diseases (e.g. diabetes, uremia, cirrhosis of the liver, hypertension); 205 had undergone semen examination before; 107 reported a duration of abstinence of <2 days or >7 days; 32 had sample spillage or failed to collect the semen samples; 71 had been diagnosed with azoospermia or cryptozoospermia; and 16 had been diagnosed with neurological or psychiatric disorders. |
| Kohn, T. P. 2017 | Men were included in this analysis if unable to achieve a pregnancy within 12 months, and had no known genetic or obstructive causes of infertility.  Other criteria were not reported. |
| Chen, Q. 2016 | The participants were recruited by the following criteria: older than 18 y; abstinence period between 2 and 7 days; sophomores studying in the University Town of Chongqing city. Those who met any of the following criteria were excluded: (1) previous diagnosis by urologist with any of the listed conditions: inflammation of urogenital system, epididymitis, testicular injury, incomplete orchiocatabasis; (2) treatment history of varicocele; (3) detected by the urologist at the field of recruitment as any of the following conditions: absence of prominentia laryngea, absence of pubes, abnormal breast, abnormal penis, absence of testis, epididymal knob or varicocele. A total of 872 males participated; 796 of them were eligible and the remaining 76 were excluded due to ineligible abstinence period (n = 33), urogenital disorders (n = 36), or failure to provide semen sample (n = 7). In June 2014, 656 of the 796 eligible subjects (82.4%) were followed up and finished all the procedures. |
| Li, Y. 2013 | Permanent male residents of Chinese Han aged 20–40 years old were eligible for the study. The exclusion criteria included: diagnosis of reproductive or urological diseases; other known reproductive disorders or an identifiable history of infertility, vasoligation or chronic diseases (diabetes, hypertension, etc.); taking medicine for physical or mental illnesses; small testis (≤12 ml); reported duration of abstinence from sexual intercourse and/or masturbation for less than 2 days or more than 7 days; and exposure to an occupation that might influence semen quality. All of the participants were informed of the purpose of the study, the requirement for 2–7 days' abstinence from sexual intercourse and/or masturbation, and the possible benefits and risks of participating in the study. |
| Liu, M. M. 2017 | The volunteers, ages 18–50 years (mean 29.56+5.38 years), were selected for this study according to the following criteria: 1) a good and regular sleep duration and ideal bedtime established over the last 6 months prior to starting the study; 2) no clinical manifestations of neurological or psychiatric disorders, and no history of trauma or surgical treatments; 3) no medical conditions in which a man does not have any measurable level of sperm in his semen or there is no sperm in the ejaculate; 4) no medical problems such as chronic systemic disease, endocrine disease, urinary tract infections, or varicocele; 5) no prescribed drugs taken within 1 month prior to the study; and 6) written informed consent for using the sperm. |
