# Supplementary material for: Effects of Sleep Disorders and Circadian Rhythm Changes on Male Reproductive Health: A Systematic Review and Meta-analysis
Source: Front Physiol. 2022 Jul 13;13:913369. doi: 10.3389/fphys.2022.913369 (PMC9326175; doi:10.3389/fphys.2022.913369)
Supplement: Supplementary file 2 [file DataSheet1.docx]

**Appendix 1.Pubmed search strategy**

**Searched February 24, 2022.**

#1 "Sleep"[MeSH Terms] OR "sleep disorders, circadian rhythm"[MeSH Terms] OR "Sleep Wake Disorders"[MeSH Terms] OR "Dyssomnias"[MeSH Terms] OR "Sleep Deprivation"[MeSH Terms] OR "Sleep Initiation and Maintenance Disorders"[MeSH Terms] OR "Circadian Rhythm"[MeSH Terms] OR "Chronobiology Disorders"[MeSH Terms] OR "Shift Work Schedule"[MeSH Terms] (222,149)

#2 "Sleep"[Title/Abstract] OR "sleep*"[Title/Abstract] OR "sleep disturbance"[Title/Abstract] OR "Sleep Disorders"[Title/Abstract] OR "insomnia"[Title/Abstract] OR "dyssomnia"[Title/Abstract] OR "Sleep Deprivation"[Title/Abstract] OR "circadian rhythm"[Title/Abstract] OR "sleep duration"[Title/Abstract] OR "bedtime"[Title/Abstract] OR "sleep quality"[Title/Abstract] OR "shift work"[Title/Abstract] OR "night work"[Title/Abstract] (239,779)

#3 #1 OR #2 (319,490)

#4 "Reproductive Health"[MeSH Terms] OR "Reproduction"[MeSH Terms] OR "Semen Analysis"[MeSH Terms] OR "Sperm Count"[MeSH Terms] OR "Sperm Maturation"[MeSH Terms] OR "Sperm Motility"[MeSH Terms] OR "Oligospermia"[MeSH Terms] OR "Asthenozoospermia"[MeSH Terms] OR "Azoospermia"[MeSH Terms] OR "Testosterone"[MeSH Terms] OR "Follicle Stimulating Hormone"[MeSH Terms] OR "Luteinizing Hormone"[MeSH Terms] (1,307,296)

#5 "reproductive health"[Title/Abstract] OR "reproduction"[Title/Abstract] OR "sperm count"[Title/Abstract] OR "semen parameters"[Title/Abstract] OR "sperm*"[Title/Abstract] OR "Semen*"[Title/Abstract] OR "semen quality"[Title/Abstract] OR "reproductive hormones"[Title/Abstract] OR "follicle-stimulating hormone"[Title/Abstract] OR "FSH"[Title/Abstract] OR "luteinizing hormone"[Title/Abstract] OR "LH"[Title/Abstract] OR "testosterone"[Title/Abstract] (420,758)

#6 #4 OR #5 (1,513,840)

#7 #3 AND #6 (13,280)

#8 "Animals"[MeSH Terms] OR "review"[Publication Type] OR "review"[Title/Abstract] OR "mice"[Title/Abstract] OR "mouse"[Title/Abstract] (25,896,157)

#9 #7 NOT #8 (296)

(("Reproductive Health"[MeSH Terms] OR "Reproduction"[MeSH Terms] OR "Semen Analysis"[MeSH Terms] OR "Sperm Count"[MeSH Terms] OR "Sperm Maturation"[MeSH Terms] OR "Sperm Motility"[MeSH Terms] OR "Oligospermia"[MeSH Terms] OR "Asthenozoospermia"[MeSH Terms] OR "Azoospermia"[MeSH Terms] OR "Testosterone"[MeSH Terms] OR "follicle stimulating hormone"[MeSH Terms] OR "Luteinizing Hormone"[MeSH Terms] OR ("Reproductive Health"[Title/Abstract] OR "Reproduction"[Title/Abstract] OR "Sperm Count"[Title/Abstract] OR "semen parameters"[Title/Abstract] OR "sperm*"[Title/Abstract] OR "semen*"[Title/Abstract] OR "semen quality"[Title/Abstract] OR "reproductive hormones"[Title/Abstract] OR "follicle stimulating hormone"[Title/Abstract] OR "FSH"[Title/Abstract] OR "Luteinizing Hormone"[Title/Abstract] OR "LH"[Title/Abstract] OR "Testosterone"[Title/Abstract])) AND ("Sleep"[MeSH Terms] OR "sleep disorders, circadian rhythm"[MeSH Terms] OR "Sleep Wake Disorders"[MeSH Terms] OR "Dyssomnias"[MeSH Terms] OR "Sleep Deprivation"[MeSH Terms] OR "Sleep Initiation and Maintenance Disorders"[MeSH Terms] OR "Circadian Rhythm"[MeSH Terms] OR "Chronobiology Disorders"[MeSH Terms] OR "Shift Work Schedule"[MeSH Terms] OR ((("Sleep"[MeSH Terms] OR "sleep disorders, circadian rhythm"[MeSH Terms] OR "Sleep Wake Disorders"[MeSH Terms] OR "Dyssomnias"[MeSH Terms] OR "Sleep Deprivation"[MeSH Terms] OR "Sleep Initiation and Maintenance Disorders"[MeSH Terms] OR "Circadian Rhythm"[MeSH Terms] OR "Chronobiology Disorders"[MeSH Terms] OR "Shift Work Schedule"[MeSH Terms]) AND "Sleep"[Title/Abstract]) OR "sleep*"[Title/Abstract] OR "sleep disturbance"[Title/Abstract] OR "Sleep Disorders"[Title/Abstract] OR "insomnia"[Title/Abstract] OR "dyssomnia"[Title/Abstract] OR "Sleep Deprivation"[Title/Abstract] OR "Circadian Rhythm"[Title/Abstract] OR "sleep duration"[Title/Abstract] OR "bedtime"[Title/Abstract] OR "sleep quality"[Title/Abstract] OR "shift work"[Title/Abstract] OR "night work"[Title/Abstract]))) NOT ("Animals"[MeSH Terms] OR "review"[Publication Type] OR "review"[Title/Abstract] OR "mice"[Title/Abstract] OR "mouse"[Title/Abstract])

**Appendix 2.Embase search strategy**

**Searched February 24, 2022.**

#1 'Sleep'/exp OR 'sleep disorders, circadian rhythm'/exp OR 'Sleep Wake Disorders'/exp OR 'Dyssomnias'/exp OR 'Sleep Deprivation'/exp OR 'Sleep Initiation and Maintenance Disorders'/exp OR 'Circadian Rhythm'/exp OR 'Chronobiology Disorders'/exp OR 'Shift Work Schedule'/exp (509,512)

#2 'sleep':ab,ti OR 'sleep*':ab,ti OR 'sleep disturbance':ab,ti OR 'sleep disorders':ab,ti OR 'insomnia':ab,ti OR 'dyssomnia':ab,ti OR 'sleep deprivation':ab,ti OR 'circadian rhythm':ab,ti OR 'sleep duration':ab,ti OR 'bedtime':ab,ti OR 'sleep quality':ab,ti OR 'shift work':ab,ti OR 'night work':ab,ti (356,244)

#3 #1 OR #2 (569,382)

#4 'Reproductive Health'/exp OR 'Reproduction'/exp OR 'Semen Analysis'/exp OR 'Sperm Count'/exp OR 'Sperm Maturation'/exp OR 'Sperm Motility'/exp OR 'Oligospermia'/exp OR 'Asthenozoospermia'/exp OR 'Azoospermia'/exp OR 'Testosterone'/exp OR 'Follicle Stimulating Hormone'/exp OR 'Luteinizing Hormone'/exp (1,601,711)

#5 'reproductive health':ab,ti OR 'reproduction':ab,ti OR 'sperm count':ab,ti OR 'semen parameters':ab,ti OR 'sperm*':ab,ti OR 'Semen*':ab,ti OR 'semen quality':ab,ti OR 'reproductive hormones':ab,ti OR 'follicle stimulating hormone':ab,ti OR 'FSH':ab,ti OR 'luteinizing hormone':ab,ti OR 'LH':ab,ti OR 'testosterone':ab,ti (472,673)

#6 #4 OR #5 (1,799,800)

#7 #3 AND #6 (22,708)

#8 'animals'/exp OR 'review':it OR 'review':ab,ti OR 'mice':ab,ti OR 'mouse':ab,ti (30,866,813)

#9 #7 NOT #8 (1218)

**Appendix 3.The Cochrane library search strategy**

**Searched February 24, 2022.**

#1 MeSH descriptor:[Sleep] explode all trees(6210)

#2 MeSH descriptor:[Sleep disorders, circadian rhythm] explode all trees(206)

#3 MeSH descriptor:[Sleep Wake Disorders] explode all trees(8936)

#4 MeSH descriptor:[Dyssomnias] explode all trees(7339)

#5 MeSH descriptor:[Sleep Deprivation] explode all trees(801)

#6 MeSH descriptor:[Sleep Initiation and Maintenance Disorders] explode all trees(2690)

#7 MeSH descriptor:[Circadian Rhythm] explode all trees(3090)

#8 MeSH descriptor:[Chronobiology Disorders] explode all trees(261)

#9 MeSH descriptor:[Shift Work Schedule] explode all trees(32)

#10 (sleep):ti,ab,kw(41579)

#11 (sleep*):ti,ab,kw(44214)

#12 (sleep disturbance):ti,ab,kw(3272)

#13 (Sleep Disorders):ti,ab,kw(9760)

#14 (insomnia):ti,ab,kw(11878)

#15 (dyssomnia):ti,ab,kw(17)

#16 (Sleep Deprivation):ti,ab,kw(1838)

#17 (circadian rhythm):ti,ab,kw(5107)

#18 (sleep duration):ti,ab,kw(6455)

#19 (bedtime):ti,ab,kw(4225)

#20 (sleep quality):ti,ab,kw(15966)

#21 (shift work):ti,ab,kw(1124)

#22 (night work):ti,ab,kw(1035)

#23 #1 OR #2 OR #3 OR #4 OR #5 OR #6 OR #7 OR #8 OR #9 OR #10 OR #11 OR #12 OR #13 OR #14 OR #15 OR #16 OR #17 OR #18 OR #19 OR #20 OR #21 OR #22 (56973)

#24 MeSH descriptor:[Reproductive Health] explode all trees(94)

#25 MeSH descriptor:[Reproduction] explode all trees(25855)

#26 MeSH descriptor:[Semen Analysis] explode all trees(575)

#27 MeSH descriptor:[Sperm Count] explode all trees(348)

#28 MeSH descriptor:[Sperm Maturation] explode all trees(3)

#29 MeSH descriptor:[Sperm Motility] explode all trees(358)

#30 MeSH descriptor:[Oligospermia] explode all trees(191)

#31 MeSH descriptor:[Asthenozoospermia] explode all trees(51)

#32 MeSH descriptor:[Azoospermia] explode all trees(22)

#33 MeSH descriptor:[Testosterone] explode all trees(3113)

#34 MeSH descriptor:[Follicle Stimulating Hormone] explode all trees(2025)

#35 MeSH descriptor:[Luteinizing Hormone] explode all trees(1654)

#36 (reproductive health):ti,ab,kw(2836)

#37 (reproduction):ti,ab,kw(3672)

#38 (sperm count):ti,ab,kw(1029)

#39 (semen parameters):ti,ab,kw(754)

#40 (sperm*):ti,ab,kw(6688)

#41 (Semen*):ti,ab,kw(2363)

#42 (semen quality):ti,ab,kw(648)

#43 (reproductive hormones):ti,ab,kw(560)

#44 (follicle stimulating hormone):ti,ab,kw(3847)

#45 (FSH):ti,ab,kw(5783)

#46 (luteinizing hormone):ti,ab,kw(4027)

#47 (LH):ti,ab,kw(5414)

#48 (testosterone):ti,ab,kw(8117)

#49 #24 OR #25 OR #26 OR #27 OR #28 OR #29 OR #30 OR #31 OR #32 OR #33 OR #34 OR #35 OR #36 OR #37 OR #38 OR #39 OR #40 OR #41 OR #42 OR #43 OR #44 OR #45 OR #46 OR #47 OR #48 (50522)

#50 #23 AND #49 (1167)

#51 MeSH descriptor:[Review] explode all trees(2)

#52 (review):ti,ab,kw(68115)

#53 (mice):ti,ab,kw(5101)

#54 (mouse):ti,ab,kw(5101)

#55 #51 OR #52 OR #53 OR #54 (72940)

#56 #50 NOT #55 (1067)

**Appendix 4.Web of Science search strategy**

**Searched February 24, 2022.**

#1 TS=( "Sleep" OR "sleep disorders, circadian rhythm" OR "Sleep Wake Disorders" OR "Dyssomnias" OR "Sleep Deprivation" OR "Sleep Initiation and Maintenance Disorders" OR "Circadian Rhythm" OR "Chronobiology Disorders" OR "Shift Work Schedule" ) OR AB=( "Sleep" OR "sleep*" OR "sleep disturbance" OR "Sleep Disorders" OR "insomnia" OR "dyssomnia" OR "Sleep Deprivation" OR "circadian rhythm" OR "sleep duration" OR "bedtime" OR "sleep quality" OR "shift work" OR "night work" ) (670,255)

#2 TS=( "Reproductive Health" OR "Reproduction" OR "Semen Analysis" OR "Sperm Count" OR "Sperm Maturation" OR "Sperm Motility" OR "Oligospermia" OR "Asthenozoospermia" OR "Azoospermia" OR "Testosterone" OR "Follicle Stimulating Hormone" OR "Luteinizing Hormone" ) OR AB=( "reproductive health" OR "reproduction" OR "sperm count" OR "semen parameters" OR "sperm*" OR "Semen*" OR "semen quality" OR "reproductive hormones" OR "follicle-stimulating hormone" OR "FSH" OR "luteinizing hormone" OR "LH" OR "testosterone" ) (2,650,586)

#3 #1 AND #2 (18,768)

#4 TS=( "Animals" OR "review" ) OR AB=( "review" OR "mice" OR "mouse" ) (25,342,339)

#5 #3 NOT #4 (4,015)

**Appendix 5.Scopus search strategy**

**Searched February 24, 2022.**

#1 TITLE-ABS ( "sleep" ) OR TITLE-ABS ( "sleep*" ) OR TITLE-ABS ( "sleep disturbance" ) OR TITLE-ABS ( "Sleep Disorders" ) OR TITLE-ABS ( "insomnia" ) OR TITLE-ABS ( "dyssomnia" ) OR TITLE-ABS ( "Sleep Deprivation" ) OR TITLE-ABS ( "circadian rhythm" ) OR TITLE-ABS ( "sleep duration" ) OR TITLE-ABS ( "bedtime" ) OR TITLE-ABS ( "sleep quality" ) OR TITLE-ABS ( "shift work" ) OR TITLE-ABS ( "night work" ) (325,850)

#2 TITLE-ABS ( "reproductive health" ) OR TITLE-ABS ( "reproduction" ) OR TITLE-ABS ( "sperm count" ) OR TITLE-ABS ( "semen parameters" ) OR TITLE-ABS ( "sperm*" ) OR TITLE-ABS ( "Semen*" ) OR TITLE-ABS ( "semen quality" ) OR TITLE-ABS ( "reproductive hormones" ) OR TITLE-ABS ( "follicle stimulating hormone" ) OR TITLE-ABS ( "FSH" ) OR TITLE-ABS ( "luteinizing hormone" ) OR TITLE-ABS ( "LH" ) OR TITLE-ABS ( "testosterone" ) (712,456)

#3 TITLE-ABS ( "review" ) OR TITLE-ABS ( "mice" ) OR TITLE-ABS ( "mouse" ) OR TITLE-ABS ( "animals" ) (6,275,286)

#4 (#1 AND #2) NOT #3 (3,277)

((TITLE-ABS ( "sleep" ) OR TITLE-ABS ( "sleep*" ) OR TITLE-ABS ( "sleep disturbance" ) OR TITLE-ABS ( "Sleep Disorders" ) OR TITLE-ABS ( "insomnia" ) OR TITLE-ABS ( "dyssomnia" ) OR TITLE-ABS ( "Sleep Deprivation" ) OR TITLE-ABS ( "circadian rhythm" ) OR TITLE-ABS ( "sleep duration" ) OR TITLE-ABS ( "bedtime" ) OR TITLE-ABS ( "sleep quality" ) OR TITLE-ABS ( "shift work" ) OR TITLE-ABS ( "night work" )) AND (TITLE-ABS ( "reproductive health" ) OR TITLE-ABS ( "reproduction" ) OR TITLE-ABS ( "sperm count" ) OR TITLE-ABS ( "semen parameters" ) OR TITLE-ABS ( "sperm*" ) OR TITLE-ABS ( "Semen*" ) OR TITLE-ABS ( "semen quality" ) OR TITLE-ABS ( "reproductive hormones" ) OR TITLE-ABS ( "follicle stimulating hormone" ) OR TITLE-ABS ( "FSH" ) OR TITLE-ABS ( "luteinizing hormone" ) OR TITLE-ABS ( "LH" ) OR TITLE-ABS ( "testosterone" ))) AND NOT (TITLE-ABS ( "review" ) OR TITLE-ABS ( "mice" ) OR TITLE-ABS ( "mouse" ) OR TITLE-ABS ( "animals" ))
